# Quality of Life and Treatment Satisfaction in Patients Receiving Intravitreal Injection Therapy for Neovascular Age-Related Macular Degeneration, Diabetic Macular Edema, and Retinal Vein Occlusion: A Cross-Sectional Study

**DOI:** 10.7759/cureus.91004

**Published:** 2025-08-26

**Authors:** Konstantinos Flindris, Chrysa Chatzipetrou, Eleni Papafotiou, Athanasios Kaliardas, Ioannis Koumpoulis, Ioannis Melissourgos

**Affiliations:** 1 Ophthalmology, General Hospital of Ioannina "G. Hatzikosta", Ioannina, GRC; 2 Medical School, Aristotle University of Thessaloniki, Thessaloniki, GRC

**Keywords:** diabetic macular oedema, intravitreal (ivt) injection, neovascular age-related macular degeneration, patient-centered outcomes research, quality of life (qol), retinal vein occlusion, therapy satisfaction, wet age related macular degeneration

## Abstract

Purpose

Neovascular age-related macular degeneration (nAMD), diabetic macular edema (DME), and retinal vein occlusion (RVO) are leading causes of vision loss treated with intravitreal injections. The impact of these chronic treatments on patients’ vision-related quality of life (QoL) and treatment satisfaction (TS) across different diseases remains unclear. This study aims to evaluate and compare vision-related QoL and TS in patients with nAMD, DME, or RVO undergoing intravitreal therapy, and to identify factors associated with QoL.

Methods

Single-center cross-sectional study including 88 patients (58 with nAMD, 22 DME, 8 RVO) receiving intravitreal anti-vascular endothelial growth factor (VEGF) or corticosteroid injections (≥3 prior injections). Vision-related QoL was assessed with the National Eye Institute Visual Function Questionnaire-25 (VFQ-25), and TS was measured with the Macular Disease Treatment Satisfaction Questionnaire (MacTSQ). Best-corrected visual acuity (BCVA) of both eyes was measured (logMAR), and a complete ophthalmological examination was performed. We compared VFQ-25 and MacTSQ scores among diagnostic groups and analyzed correlations between QoL, TS, and systemic factors. Multivariable linear regression identified independent predictors of QoL.

Results

Mean VFQ-25 composite score was 65.1 ± 20.7 (out of 100), indicating moderate QoL impairment, with no significant difference between nAMD, DME, and RVO groups (p = 0.40). Mean MacTSQ score was 81.9 ± 13.5, reflecting high treatment satisfaction, also with no difference among diagnoses (p = 0.14). Worse BCVA (higher mean logMAR of both eyes) and older age were each significantly associated with lower QoL (Spearman r ≈ -0.50 and r ≈ -0.48; p < 0.001). In multivariable analysis, older age (p = 0.004), presence of dyslipidemia (p = 0.03) or depression (p = 0.04), and worse BCVA (p < 0.001) were independent predictors of lower VFQ-25 scores. Diagnostic group, bilateral treatment, and other comorbidities were not independent predictors of QoL. Treatment satisfaction was uniformly high across all groups and was not significantly correlated with age or QoL.

Conclusions

Patients with nAMD, DME, and RVO receiving intravitreal injections report similar levels of vision-specific QoL impairment and high satisfaction with treatment. Preservation of visual acuity is the key determinant of better QoL, underscoring the importance of effective therapy and adherence. Additionally, older age and systemic factors such as dyslipidemia and depression adversely affect QoL, highlighting the need for a multidisciplinary management approach. These findings suggest that regardless of retinal disease etiology, maintaining vision and addressing comorbidities are critical to optimizing patient-centered outcomes.

## Introduction

Age-related macular degeneration (AMD), diabetic retinopathy including diabetic macular edema (DME), and retinal vein occlusion (RVO) are among the leading causes of vision loss worldwide in older adults and the working-age population [1]. Neovascular (“wet”) AMD (nAMD) is one of the leading causes of irreversible blindness in the elderly, especially in developed countries [2]. Diabetic retinopathy and its complication DME represent a primary cause of vision impairment among working-age adults globally [3]. RVO, which includes branch and central retinal vein occlusions, is the second most common retinal vascular disorder causing vision loss after diabetic retinopathy, affecting an estimated 16-28 million people worldwide [4]. These retinal conditions all primarily affect the macula, leading to central visual acuity loss, metamorphopsia, and other visual deficits that can severely impact patients’ daily functioning and quality of life (QoL) [5].

The advent of intravitreal pharmacotherapy has revolutionized the management of these diseases. In particular, repeated intravitreal injections of anti-vascular endothelial growth factor (anti-VEGF) agents have become the standard of care for nAMD, DME with center-involving macular edema, and RVO-associated macular edema [6]. Anti-vascular endothelial growth factor (VEGF) and corticosteroid intravitreal injections therapy can stabilise or improve vision in a significant proportion of patients, markedly improving visual prognosis. Consequently, the prevalence of severe visual impairment from these clinical conditions has declined in regions where anti-VEGF therapy is widely used [7]. However, these benefits come at a cost, as patients often require frequent clinic visits and recurrent injections, as often as every 4-8 weeks, to maintain vision gains. The continuous treatment and monitoring regimen can impose substantial burdens on patients and caregivers, causing anxiety, stress, and depression. In real-world practice, patients often express both gratitude for preserved vision and frustration with the chronic nature of therapy, including fear of injections and logistical challenges of frequent appointments [8].

In chronic eye diseases, patient-reported outcomes such as vision-related QoL and treatment satisfaction (TS) are crucial for comprehensive care evaluation. Previous studies have shown that vision-related QoL in patients with nAMD, DME or RVO-associated macular edema is closely linked to visual acuity - patients with better preserved vision tend to report higher QoL scores [9]. Moreover, comorbidities can modulate QoL; for example, depression is commonly associated with vision loss in nAMD and can further reduce QoL scores and hinder therapy adherence [10]. However, there is limited data directly comparing patient-reported QoL and TS across different retinal diseases under intravitreal injection therapy. It is not well established whether patients with AMD, DME, and RVO experience different levels of QoL impairment or TS, or whether the intravitreal therapy burden yields a similar patient experience across these conditions.

Herein, this study aimed to investigate vision-related QoL and TS in a group of patients receiving intravitreal injection therapy for nAMD, DME, or RVO-associated macular edema in a single center. Our objectives were: (1) to compare QoL scores and TS scores between the three diagnostic groups; (2) to examine the relationships between QoL, visual acuity, and age; and (3) to identify demographic or clinical factors associated with poorer QoL. By providing insight into patients’ perspectives, the findings can guide comprehensive management strategies to maintain vision through therapy adherence but, also, QoL in those undergoing long-term intravitreal therapy.

## Materials and methods

This study was a single hospital-based cross-sectional study of patients with nAMD, DME, or RVO-associated macular edema who were receiving either anti-VEGF or corticosteroid intravitreal injection therapy. The study was conducted at a single tertiary Ophthalmology Department of General Hospital of Ioannina “G. Hatzikosta” from July 2024 to May 2025. Consecutive eligible patients presenting to the ophthalmology department during this period were invited to participate. Inclusion criteria were: diagnosis of nAMD, DME, or macular edema due to RVO (branch or central) in at least one eye; currently undergoing intravitreal injection treatment (anti-VEGF agents and/or corticosteroid implants as per standard of care); history of at least three prior intravitreal injections (to ensure participants had sufficient treatment experience); age 18 or older; and ability to complete the questionnaires via interview in the native language.

Exclusion criteria included the presence of other significant ocular pathology that could confound vision-related QoL, such as advanced glaucoma, visually significant cataract, ocular trauma, corneal opacities, or other retinal diseases (e.g., retinitis pigmentosa). Patients with cognitive impairment or any condition precluding informed consent or valid questionnaire responses were also excluded.

All participants underwent a complete ophthalmologic examination on the day of survey administration. Best-corrected visual acuity (BCVA) was measured for each eye using Early Treatment Diabetic Retinopathy Study (ETDRS) charts at 4 meters. The letter score was recorded and converted to the logarithm of the minimal angle of resolution (logMAR) for analysis. For the purposes of correlating vision with QoL, the mean logMAR of both eyes was considered the primary visual acuity metric. Intraocular pressure was measured by Goldmann applanation tonometry. A slit-lamp biomicroscopic examination of the anterior segment was performed, and dilated fundus examination was conducted with a slit-lamp lens to assess the macula and optic disc. Optical coherence tomography (OCT) was obtained for each eye to document central retinal thickness and confirm the presence of macular edema or subretinal fluid as relevant to the underlying disease.

In addition, optical coherence tomography angiography (OCTA) was conducted to visualize chorioretinal neovascular membranes in AMD or areas of nonperfusion in diabetic retinopathy/RVO. Key clinical information (diagnosis, duration of disease, number of prior injections, current treatment regimen, and ocular comorbidities) was obtained from medical records and baseline demographic data were recorded, including age, sex, laterality of disease (one eye vs both eyes requiring treatment), and systemic comorbidities (hypertension, dyslipidemia, diabetes mellitus (DM) (for AMD/RVO patients without DME), cardiovascular disease (e.g. history of coronary artery disease or stroke), and depression or other psychiatric illness by self-report or medical record).

Vision-related QoL was assessed using the interviewer-administered National Eye Institute Visual Function Questionnaire-25 (NEI VFQ-25). NEI VFQ-25 is widely used in research on retina diseases and is sensitive to changes in visual acuity, making it a useful tool for assessing the functional impact of treatments. This instrument includes 25 items covering domains such as general vision, near and distance activities, social functioning, mental health related to vision, role limitations, dependency, driving, color vision, and peripheral vision. An additional general health rating item was included per standard VFQ-25 administration, but the general health item was not used in computing the vision-specific composite score. Each NEI VFQ-25 item response was converted to a 0-100 Likert-scale, and domain scores and an overall composite score were calculated according to the NEI VFQ-25 scoring guidelines. The composite score represents the average of all vision-targeted subscale scores, with higher scores indicating better vision-related QoL (maximum 100).

The questionnaire was administered in the patients’ native language by a trained interviewer who read the questions and recorded responses, to accommodate any patients with low vision or literacy issues. Permission to use the NEI VFQ-25 was obtained from the rights holders prior to study initiation [11]. Interviews were conducted in a private, quiet space to ensure patient comfort and candor.

Treatment satisfaction was evaluated with the Macular Disease Treatment Satisfaction Questionnaire (MacTSQ). The MacTSQ is a validated disease-specific questionnaire for assessing satisfaction with treatment among patients with retina conditions affecting the macula. It consists of 13 items that address various aspects of treatment experience, including convenience of the treatment schedule, understanding of the treatment, side effects, and perceived effectiveness. Each item is rated on a Likert-scale response (scored 0 to 6), and item scores are summed to produce a total score ranging from 0 to 100, with higher scores denoting greater TS. As with the VFQ-25, the MacTSQ was administered in a professionally translated version via interview. Permission to use the MacTSQ was obtained from the rights holders prior to study initiation [12]. The interviewers ensured that patients understood each question and provided clarification if needed, without influencing their response.

Together, NEI VFQ-25 and MacTSQ provide complementary information: the former reflects the patient’s perceived visual ability and well-being, while the latter reflects their contentment with the process and outcomes of treatment.

All data were entered into a secure database and analyzed using SPSS Statistics version 29 (IBM Corp., Armonk, USA). Descriptive statistics were computed for key variables. Mean and standard deviation (SD) were calculated for continuous variables, and frequencies and percentages for categorical variables. Continuous variables were assessed for normality using the Shapiro-Wilk test and Q-Q plots. The NEI VFQ-25 composite scores were approximately normally distributed, whereas the MacTSQ total scores showed a skewed distribution, since many patients reported high satisfaction, leading to a ceiling effect. Thus, parametric tests were used for analyses involving VFQ-25 scores, and non-parametric tests were used for MacTSQ scores.

Patient demographic and clinical characteristics were summarized by diagnostic group (nAMD, DME, RVO), including mean age, gender distribution, proportion with bilateral disease, logMAR in the better eye, and prevalence of key comorbidities in each group. Independent sample t-tests and Mann-Whitney tests, depending on data distribution, were used to evaluate the possible impact of the above baseline characteristics and underlying conditions on QoL and TS.

To compare vision-related QoL between the three disease groups, one-way analysis of variance (ANOVA) was performed on the VFQ-25 composite scores, with disease diagnosis as the independent factor. Post hoc comparisons with Bonferroni adjustment were planned if any overall difference was detected. For TS scores, the Kruskal-Wallis test was used to compare the three groups, given the non-normal distribution. If the Kruskal-Wallis test was significant, Dunn’s post hoc pairwise comparisons (with Bonferroni correction) were conducted. Correlations between QoL and clinical variables (e.g., age, logMAR, TS score) and correlations between TS and clinical variables (e.g., age, logMAR, QoL score) were assessed using Spearman’s rank correlation (r). Correlation coefficients (r) were interpreted in terms of magnitude (with 0.1-0.3 indicating weak, 0.3-0.5 moderate, and >0.5 strong correlation).

To identify independent predictors of vision-related QoL, a multivariable linear regression analysis was performed with the VFQ-25 composite score as the dependent variable with an enter selection process. Based on clinical relevance and univariate analyses, we included the following predictors in the model: age (years), gender, bilateral treatment status (bilateral vs unilateral disease), diagnosis group (indicator variables for DME and RVO, with AMD as reference, to check if diagnosis per se influenced QoL), BCVA (mean logMAR of both eyes), and major systemic comorbidities (hypertension, dyslipidemia, DM, cardiovascular disease, and depression - each coded as binary present/absent). All variables were initially included, and at each iteration, the variable with the highest p-value was removed. The process continued until only variables with p<0.05 remained in the final model. Results were reported as regression coefficients (β) with corresponding 95% confidence intervals (CI). All hypothesis tests were two-sided, with p < 0.05 considered statistically significant.

This study adhered to the tenets of the Declaration of Helsinki and was approved by the Institutional Review Board of the host hospital’s ethics committee (Protocol ID: 10774, Date of Approval: 10-07-2024). Written informed consent was obtained from all participants prior to enrollment. Patients were assured that their decision to participate or decline would not affect their clinical care. Interviews were conducted in a manner that safeguarded privacy and encouraged honest responses. To minimize respondent burden, questionnaires were administered in a single session, taking approximately 15-20 minutes, with short breaks allowed if needed for fatigue. The study questionnaires (VFQ-25 and MacTSQ) are established instruments; permission was obtained to use and translate them as required.

All collected data were de-identified to maintain confidentiality. Participants who were found to have significant depressive symptoms or distress during the interview were offered referral for psychosocial support as part of ethical care, though formal depression screening was not a study procedure. The results of this research will be made available to participants upon request.

## Results

A total of 88 patients met the inclusion criteria during the study period and agreed to participate. The majority of patients included in the study were diagnosed with nAMD (n=58, 65.9%), followed by DME (n=22, 25%), and RVO-associated macular edema (n=8, 9.1%). The overall mean age of the participants was 75.16 ± 10.61 years, ranging from 50 to 87 years old, and 67% were male (n=59). Patients with nAMD were older on average (78.93 ± 8.71 years) than those with DME (69.27 ± 8.35 years) or RVO (64 ± 14.27 years). The mean number of intravitreal injections received was 9.18 ± 10.39, with a range of three to 74 injections, without a significant difference between nAMD and DME groups (treat-and-extend protocol after initial loading doses), and RVO patients were generally in the first year of treatment.

The distribution of anti-VEGF agents at the time of study was: ranibizumab (49%), aflibercept (30%), faricimab (15%), and dexamethasone implant or other therapy (6%). The mean BCVA of both eyes in the overall analysis was logMAR=0.396 ± 0.30, and the mean number of letters in the ETDRS chart was 33.7 ± 15.03. Overall, 24 patients (27.35%) were receiving bilateral intravitreal injections. Among the 58 nAMD patients, 11 (19%) had bilateral neovascular/exudative involvement requiring injections in both eyes. In the DME group, all 22 had type 2 DM and center-involving macular edema; 12 of these (54.5%) had bilateral DME with both eyes treated. In the RVO group (five branch RVO, three central RVO), one patient (12.5%) had bilateral disease (Table 1).

**Table 1:** Demographic and clinical characteristics of the participants nAMD: neovascular Age-related Macular Degeneration, DME: Diabetic Macular Edema, RVO: Retinal Vein Occlusion, SD: Standard Deviation,

Variable	nAMD	DME	RVO	Total
Number of patients n (%)	58 (65.9%)	22 (25%)	8 (9.1%)	88 (100%)
Male n (%)	43 (74.1%)	13 (59.1%)	3 (37.5%)	59 (67%)
Female n (%)	15 (25.9%)	9 (40.9%)	5 (62.5%)	29 (33%)
Mean age in years (± SD)	78.93 ± 8.71	69.27 ± 8.35	64 ± 14.27	75.16 ± 10.61
Mean duration of treatment in years (± SD)	2.26 ± 2.50	2.64 ± 2.17	2.0 ± 1.93	2.33 ± 2.36
Mean number of intravitreal injections (± SD)	8.72 ± 11.12	11.68 ± 9.61	5.63 ± 4.69	9.18 ± 10.39
Treatment of one eye n (%)	47 (81%)	10 (45.5%)	7 (87.5%)	64 (72.7%)
Treatment of both eyes n (%)	11 (19%)	12 (54.5%)	1 (12.5%)	24 (27.3%)

Systemic comorbidities were prevalent. Hypertension was the most common comorbidity (n = 69, 78.4%), followed by dyslipidemia (n = 59, 67%). By design, all DME patients had DM; additionally, four nAMD and two RVO patients had type 2 DM, bringing the total with DM to 38 (43.2%). Cardiovascular disease (defined as a history of coronary artery disease, myocardial infarction, stroke, and/or peripheral arterial disease) was present in 46 patients (52.3%). Notably, 27 patients (30.7%) had a documented history of depression or were under treatment for depression/anxiety. The AMD group had the highest number of depressed patients (19 out of 58, 33%), while the DME group had six patients (27%) with depression, and the RVO group had two (25%). There were no significant differences between the three diagnosis groups in terms of gender, hypertension, dyslipidemia, cardiovascular disease, or depression prevalence (p > 0.05), aside from the expected difference in DM status (Table 2).

**Table 2:** Systemic comorbidities of the participants

Variable	Yes n (%)	No n (%)
Systemic Comorbidities	84 (95.5%)	4 (4.5%)
Hypertension	69 (78.4%)	19 (21.6%)
Dyslipidemia	59 (67%)	29 (33%)
Diabetes mellitus	38 (43.2%)	50 (56.8%)
Cardiovascular disease	46 (52.3%)	42 (47.7%)
Depression	27 (30.7%)	61 (69.3%)

The overall mean NEI VFQ-25 composite score for the 88 patients was 65.06 ± 20.72, which is indicative of moderate impairment in vision-related QoL. In our study, individual composite scores ranged widely from 10 (reflecting very poor perceived visual function) to 95.83 (near-perfect QoL), underscoring the heterogeneity in visual experiences even among treated patients. When comparing by diagnostic group, nAMD patients had a mean composite score of 63.8 ± 22.26, DME patients 64.98 ± 18.49, and RVO patients 74.34 ± 12.77. There were no statistically significant differences in QoL score between the three diagnostic groups (F=0.907, p=0.407, ANOVA).

The mean MacTSQ total score was 81.94 ± 13.50, with a range of 30.59 to 100.0. The MacTSQ results indicated generally high satisfaction with treatment across the board. The distribution was left-skewed (clustered toward the higher end), which is why non-parametric tests were used. Furthermore, there were no statistically significant differences in TS score between the three diagnostic groups (H=3.913, p=0.141, Kruskal-Wallis test).

**Figure 1:**
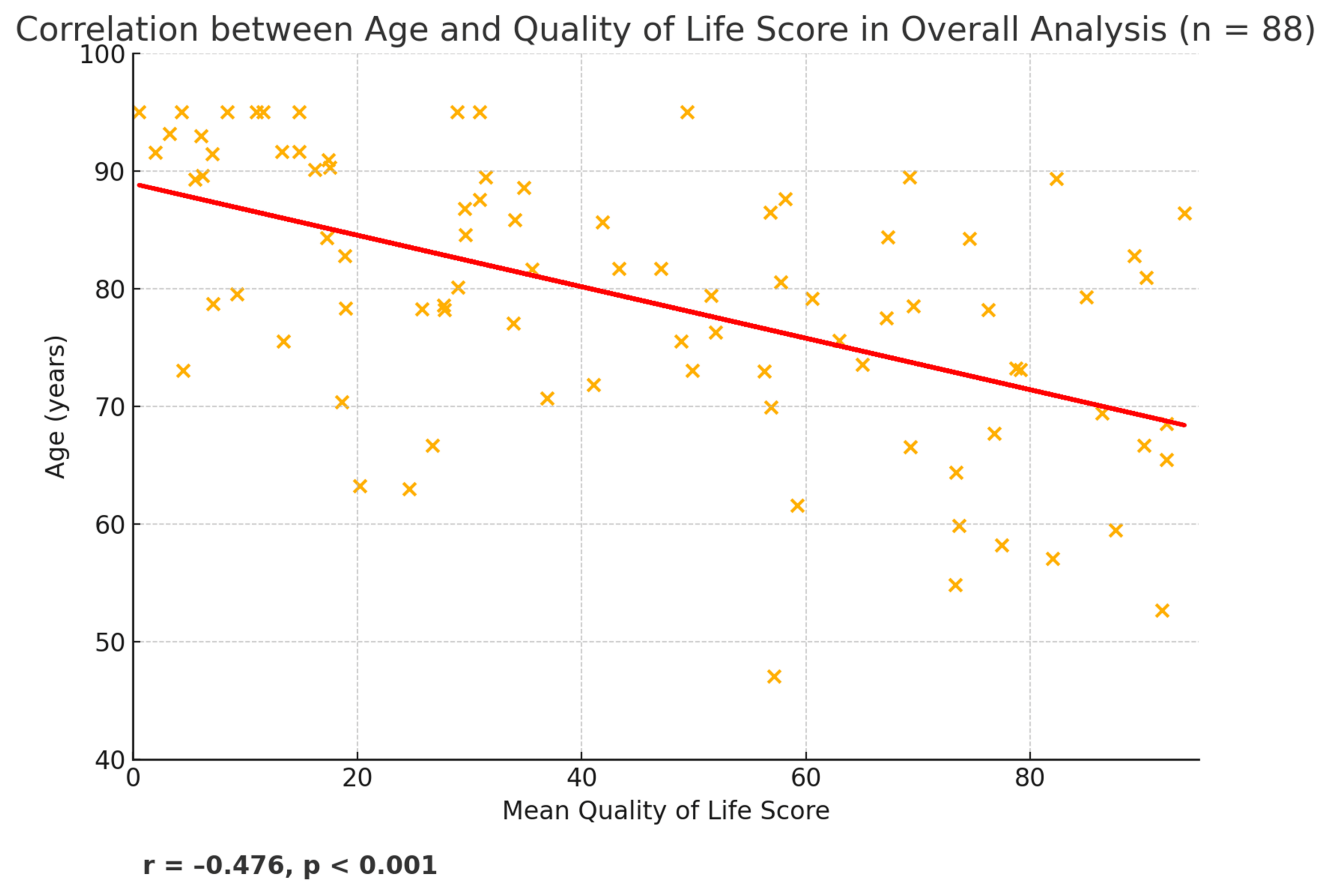
Scatter plot presenting the correlation between age and mean QoL score in overall analysis. The red line is the best-fit regression line, and the annotation shows r = –0.476, p < 0.001, reflecting a moderate negative association. QoL: Quality of Life

**Figure 2:**
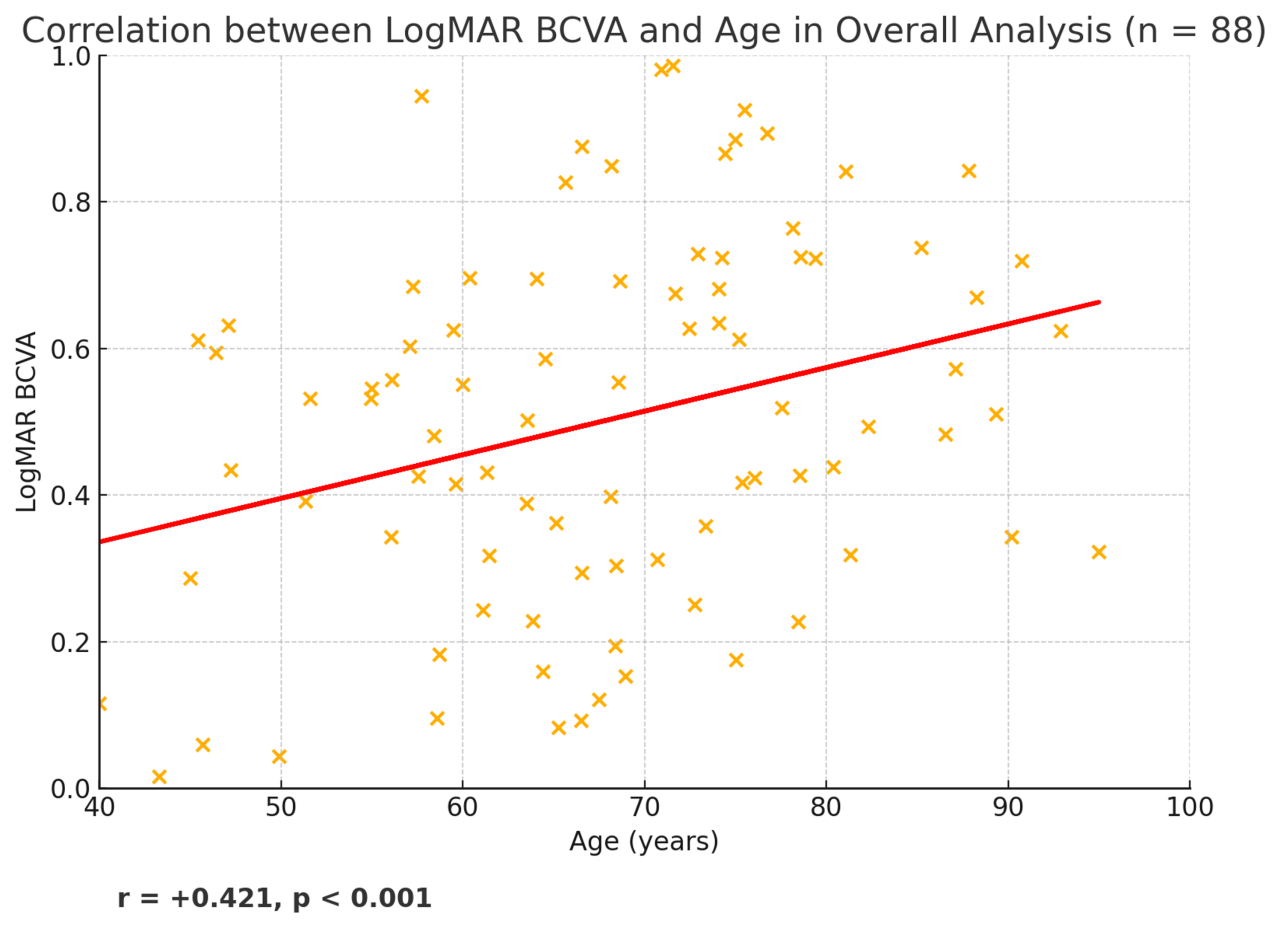
Scatter plot presenting the correlation between age (years) and mean logMAR BCVA of both eyes in overall analysis. The red line is the best-fit regression line, and the annotation shows r = +0.421, p < 0.001, reflecting a moderate positive association. BCVA: best-corrected visual acuity

Significant correlations between vision-related QoL and key variables of interest were observed. Firstly, there was a moderate negative correlation between mean QoL score and age (r = -0.476, p < 0.001) (Figure 1). Secondly, age presented a positive correlation with logMAR BCVA (r = +0.421, p < 0.001), meaning older patients generally had worse vision (higher LogMAR) (Figure 2).

**Figure 3:**
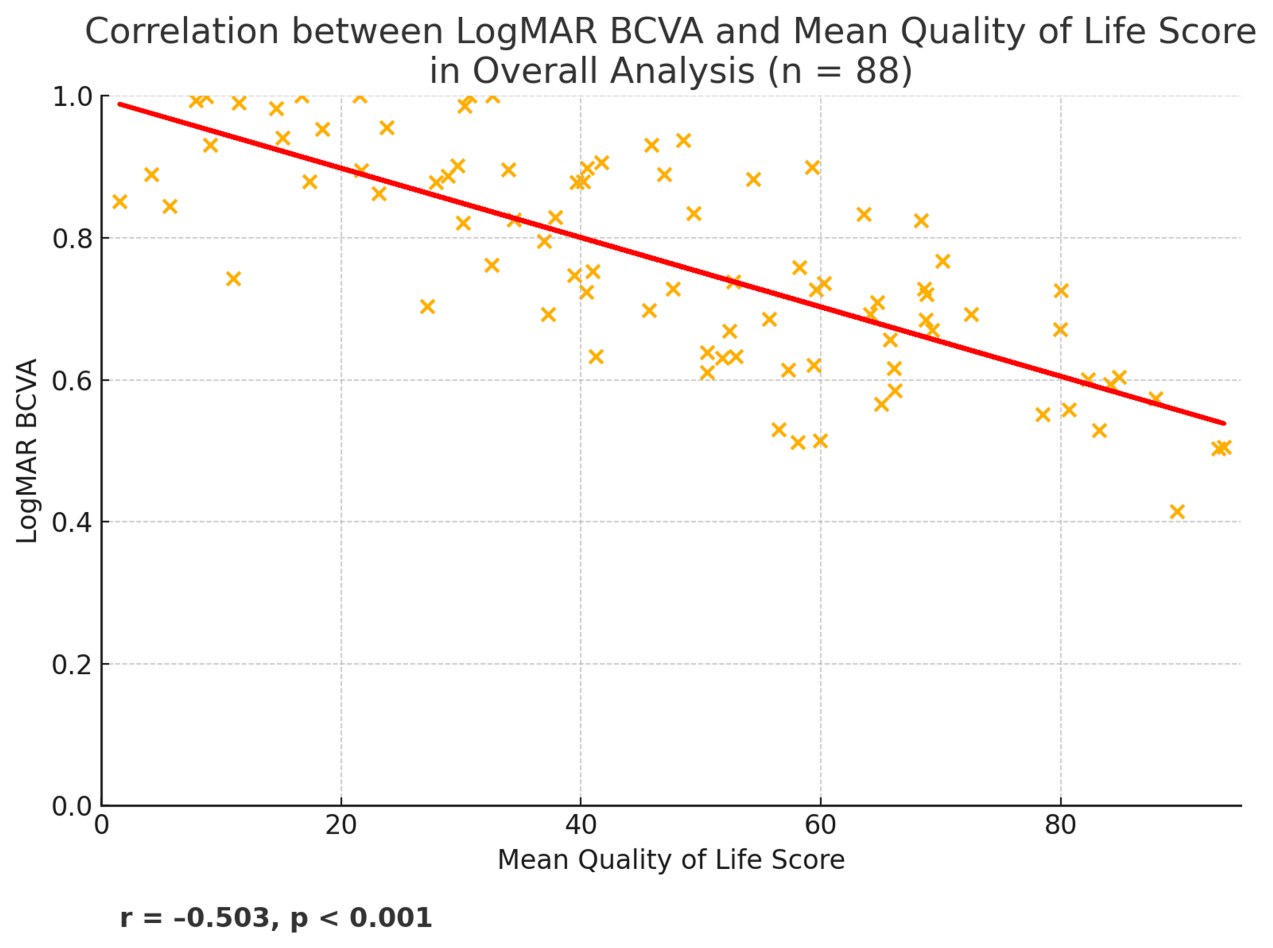
Scatter plot presenting the correlation between mean QoL score and mean logMAR BCVA of both eyes in overall analysis. The red line is the best-fit regression line, and the annotation shows r = –0.503, p < 0.001, reflecting a negative association. BCVA: Best-Corrected Visual Acuity, QoL: Quality of Life

Crucially, the mean logMAR BCVA of both eyes was related to the mean QoL score in the overall analysis (r = -0.503, p < 0.001) (Figure 3). This suggests that patients with better vision (lower LogMAR) had substantially higher QoL scores. No statistically significant association was revealed in the correlation between mean QoL score and mean TS score (p = 0.381), or between mean TS score and age in years (p = 0.908).

Further tests were conducted to assess the contribution of comorbidities to mean QoL and TS scores. Hypertension, dyslipidemia, DM, and cardiovascular disease presented a statistically significant association with mean QoL score, while only DM showed a statistically significant association with mean TS score (Table 3).

**Table 3:** Association between patient characteristics and mean QoL – TS scores QoL: Quality of Life, TS: Treatment Satisfaction *t-test, ** Mann-Whitney U test

Variable	Mean QoL score (p-value)	Mean TS score (p-value)
Sex	U= 815 (p=0.719)**	U=817.5 (p=0.736)**
Bilateral Therapy	t=0.231 (p=0.818)*	U=681 (p=0.415)**
Systemic disease	t=-0.593 (p=0.555)*	U=99 (p=0.167)**
Hypertension	U=440 (p=0.029)**	U=645 (p=0.919)**
Dyslipidemia	U=553 (p=0.007)**	U=715 (p=0.212)**
Diabetes Mellitus	t=2.67 (p=0.008)*	U=680 (p=0.023)**
Cardiovascular disease	t=2.22 (p=0.031)*	U=928.5 (p=0.754)**
Depression	U=692 (p=0.234)**	U=779.5 (p=0.690)**

To disentangle the contributions of various factors to QoL, we built a multivariable linear regression model for the VFQ-25 composite score. In the multivariable analysis, initially, all candidate predictors were entered into the model with an enter selection procedure. At each iteration, the variable with the highest p-value was removed until only variables with p < 0.05 remained in the final model. This process yielded an ultimate model with four significant independent predictors of QoL: age, dyslipidemia, depression, and BCVA (mean logMAR of both eyes) (p < 0.05 for all). The final regression equation was:

QoL = 124.374 - 0.545 × Age (years) - 8.180 × Dyslipidemia (Yes=1, No=0) - 7.289 × Depression (Yes=1, No=0) - 26.432 × mean LogMAR (both eyes).

This indicates that older age, the presence of dyslipidemia or depression, and worse visual acuity are each associated with significantly lower QoL under intravitreal injection therapy. Specifically, each additional year of age was associated with a 0.545-point decrease in the QoL score (β = -0.545, 95% CI= (-0.909, -0.181), p=0.004). Having dyslipidemia (β = -8.180, p = 0.03) or depression (β = -7.289, p = 0.04) corresponded to an 8.18-point and 7.29-point lower QoL score, respectively. Furthermore, a 1.0 increase in mean LogMAR (reflecting poorer vision) predicted a 26.43-point reduction in QoL (β = -26.432, p < 0.001). All of these predictors retained in the final model were statistically significant (p < 0.05).

Interestingly, the diagnostic group (AMD vs DME vs RVO) did not remain in the model, indicating no inherent QoL disadvantage or advantage for any particular disease when adjusting for age, vision, and comorbidities. Likewise, gender, bilateral treatment status, and other comorbidities (hypertension, DM, cardiovascular disease) were not significant independent predictors once the above factors were accounted for. The final regression model explained approximately 42% of the variance in QoL scores (adjusted R² = 0.42), which is respectable for QoL data.

## Discussion

This cross-sectional study provides a comprehensive evaluation of patient-centered outcomes in individuals receiving intravitreal injection therapy for three major retinal diseases (nAMD, DME, and RVO). Our findings highlight that vision-related QoL is moderately reduced in nAMD, DME, and RVO patients under treatment with intravitreal injections, with no significant differences between these conditions. Also, TS is high across all groups, suggesting that patients generally feel positive about their intravitreal therapy despite its burdens, while better visual acuity is strongly associated with better QoL, underscoring the paramount importance of vision preservation. Finally, systemic factors such as age, dyslipidemia, and depression have a notable adverse impact on QoL independent of vision, while the diagnostic condition of the patient (nAMD, DME, and RVO) plays no important role in QoL.

Our study is among the few to directly compare patient-reported outcomes across nAMD, DME, and RVO in the context of active intravitreal injection treatment. Previous research has often focused primarily on single conditions. For example, in nAMD, it has been documented that anti-VEGF therapy can stabilize or modestly improve VFQ-25 scores over time [13]. In our cross-sectional study, nAMD patients’ mean VFQ-25 score of ~64 is similar to baseline values reported in some nAMD populations starting treatment, which often range from 60 to 75 depending on baseline vision [14]. The DME group’s mean VFQ-25 score (~65) also aligns with prior DME studies, reporting VFQ-25 score improvement reaching up to 70 after treatment from a baseline of 60 [15]. We infer that many of our patients, across diagnoses, have achieved significant visual improvement with treatment, resulting in higher QoL levels.

Crucially, we did not detect a QoL difference between diseases. This is consistent with a real-world study, which noted that nAMD and DME patients had comparable NEI VFQ-25 scores when on chronic anti-VEGF therapy [16]. Our findings suggest that RVO patients under intravitreal injection therapy are in the same range. The implication is that the impact of these diseases on patients’ lives may be more uniform than previously thought when managed with current therapies. Any differences in QoL appear to be offset by those systemic factors (age, vision, dyslipidemia, depression) rather than the disease itself. From a clinician’s perspective, this suggests that managing to maximize vision is universally important, and older patients might need extra support because of age and comorbidities, not simply because of the diagnosis of the retinal disease and its progression [17].

In terms of TS, our findings of high satisfaction are encouraging. They resonate with prior observations that most patients are satisfied with anti-VEGF injections, especially when they appreciate that the treatment is preventing vision loss [18]. A study of nAMD patients using the MacTSQ reported a mean score of ~53 and noted that mental health status was a key determinant of satisfaction [19]. In our study, the mean score of ~82/100 is high, implying that patients may have become more accustomed to the treatment, and clinicians may have optimized injection protocols and patient education, leading to improved satisfaction and enhanced therapy adherence. Additionally, innovations like improved anesthetic techniques and clinic flow might have reduced discomfort and waiting times, thereby increasing satisfaction [20].

The lack of difference across diseases indicates that the injection experience (frequency, expectations, outcomes) is fairly similar, whether one is being treated for nAMD, DME, or RVO. The introduction of newer therapies (e.g., faricimab, with potentially longer dosing intervals) may, in the future, improve satisfaction by lessening visit frequency [21]. However, our data suggest current regimens are well-tolerated from the patient perspective.

The prominent role of visual acuity in QoL underscores that achieving and maintaining the best possible vision in both eyes should remain the primary goal in managing these conditions. Every letter gained or lost can translate to meaningful differences in a patient’s daily life. Thus, adherence to treatment schedules, prompt identification of disease recurrence through treat and extend protocol, and possibly switching therapies in suboptimal responders are all critical to optimize vision [22]. In practical terms, communicating to patients that continuing regular injections can preserve not just their sight but also their independence and lifestyle may motivate adherence [23].

Our study also highlights the need to manage systemic comorbidities and psychosocial factors. The independent negative impact of depression on QoL in these patients cannot be overstated. This aligns with literature that calls for a multidisciplinary approach in patients under intravitreal injection therapy [23]. Routine screening for depression and anxiety in retinal clinics, using simple questionnaires or probing questions, could identify vulnerable patients who might benefit from counseling, support groups, or psychiatric referral. There is evidence that treating depression in visually impaired patients can improve their functional outcomes and QoL and improve their adherence to the intravitreal injection schedule [24]. Similarly, the association of dyslipidemia with lower QoL suggests that patients with a heavy vascular risk profile might have overall worse health and functioning.

Close collaboration with primary care to ensure optimal management of blood pressure, lipids, and DM could potentially improve not only systemic health but also ocular outcomes [25]. Additionally, it points to a broader need: helping patients manage multiple medical issues. A patient overwhelmed by comorbidities may feel less capable of engaging in vision rehabilitation activities or may have less energy to overcome visual challenges, thus perceiving lower QoL [26]. Notably, age as a predictor reminds us that older patients might require additional assistance. For instance, older patients living alone might benefit from low-vision aids or occupational therapy and social support to maximize their functioning, beyond what injections can do. Our data show that age had nearly as large an effect as depression or dyslipidemia on QoL in the model, meaning that it presents a substantial factor.

Qualitative feedback from patients reveals specific annoyances such as injection discomfort or travel burden. Efforts such as extending injection intervals with newer drugs or providing injection services closer to patients' homes could further alleviate the treatment burden [27]. It’s also possible that some patients maintain high satisfaction because they have accepted the chronic nature of therapy as necessary, but as new therapies (e.g., port delivery systems or gene therapies) arise that reduce injection frequency, satisfaction could improve even more [28]. Monitoring patient satisfaction over time will be important as the treatment landscape evolves.

Key strengths of our study include the use of well-validated instruments (VFQ-25 and MacTSQ) to capture patient-reported outcomes, and the inclusion of three major retinal diseases for direct comparison under a uniform treatment setting. All patients were managed at the same institution with similar protocols, which reduces confounding due to differences in care patterns. We achieved a high participation rate, which minimizes selection bias. Additionally, we collected detailed clinical data, allowing multivariable adjustment to tease apart interrelated factors. The timing of the study also provides a contemporary view of patient experiences in the era of mature anti-VEGF therapy, which is valuable given improvements in management over time.

However, several limitations should be considered. First, the cross-sectional design captures a single time point and cannot determine causality or changes over time. We cannot discern, for example, how QoL might improve or decline with continued treatment or disease progression from baseline, nor can we definitively say treating systemic comorbidities will improve QoL scores. Further longitudinal studies are needed to observe temporal dynamics and to confirm that the independent factors we found are predictive. Second, the sample size, particularly for the RVO subgroup (n = 8), was relatively small. This limits the power to detect differences involving the RVO group and might have missed small effect sizes. The overall sample of 88, while moderate, could also limit the detection of subtle differences in TS or specific QoL subscales. Third, all patients were from a single tertiary care hospital, which may limit generalizability.

The patient population characteristics (e.g., distribution of comorbidities, health literacy, cultural attitudes) and the healthcare setting (access to treatment, cost factors) may differ in other regions or community practices. Additionally, since these were established patients on treatment, our results do not address patients who discontinue therapy or who refuse treatment - those individuals might have very different QoL outcomes. There is a potential bias from patients who found treatment intolerable or ineffective and may have dropped out of therapy. Last but not least, another limitation is that the diagnosis of some comorbidities, like depression, was based on medical history. It’s possible some patients had undiagnosed depression, or conversely, some on record may have been in remission. Despite these limitations, the study provides valuable insights, but findings should be interpreted in context and verified by further research.

Future research with prospective studies with a larger sample size is needed to evaluate how QoL and TS evolve with treatment over the years. In addition, interventional studies to address the modifiable factors - such as providing depression counseling or low-vision services - could test whether those improve patient outcomes in combination with medical treatment. From a broader perspective, investigating caregiver burden and satisfaction could be valuable, as many of these patients rely on family for transportation and care. Our study focused on patients themselves, but caregiver experience is an important facet recognized in recent literature.

## Conclusions

In conclusion, intravitreal therapies for macular diseases not only preserve vision but are generally associated with favorable patient-perceived outcomes. Patients with nAMD, DME, and RVO share a common journey of navigating life with a chronic retinal condition and frequent treatments. Our study highlights that this journey, while challenging, can be supported effectively to maintain a reasonably good QoL and high TS. From a practical standpoint, our findings underscore that clinicians should monitor not only the clinical indicators of disease control but also inquire about their patients’ daily visual function and treatment satisfaction. Although overall the treatment paradigm is well-accepted, continued efforts to reduce treatment burden (e.g., longer-acting therapies, personalized injection intervals) could further enhance patient satisfaction and adherence. By focusing on maintaining visual acuity and attending to the whole patient, clinicians can help ensure that the promise of sight-saving therapies translates into improved quality of life for those we serve. Future research should continue to explore patient-centered outcomes and interventions that further enhance the experience of patients with chronic retinal diseases.

## References

[1] Gale RP, Finger RP, Eldem B (2023). The management of neovascular age-related macular degeneration: a systematic literature review of patient-reported outcomes, patient mental health and caregiver burden. Acta Ophthalmol.

[2] Deng Y, Qiao L, Du M, Qu C, Wan L, Li J, Huang L (2022). Age-related macular degeneration: epidemiology, genetics, pathophysiology, diagnosis, and targeted therapy. Genes Dis.

[3] Ammar MJ, Hsu J, Chiang A, Ho AC, Regillo CD (2020). Age-related macular degeneration therapy: a review. Curr Opin Ophthalmol.

[4] Al-Zamil WM, Yassin SA (2017). Recent developments in age-related macular degeneration: a review. Clin Interv Aging.

[5] Senra H, Ali Z, Balaskas K, Aslam T (2016). Psychological impact of anti-VEGF treatments for wet macular degeneration-a review. Graefes Arch Clin Exp Ophthalmol.

[6] DeAngelis MM, Owen LA, Morrison MA (2017). Genetics of age-related macular degeneration (AMD). Hum Mol Genet.

[7] Fabre M, Mateo L, Lamaa D (2022). Recent advances in age-related macular degeneration therapies. Molecules.

[8] Spooner KL, Guinan G, Koller S, Hong T, Chang AA (2019). Burden of treatment among patients undergoing intravitreal injections for diabetic macular oedema in Australia. Diabetes Metab Syndr Obes.

[9] Ehlken C, Ziemssen F, Eter N, Lanzl I, Kaymak H, Lommatzsch A, Schuster AK (2020). Systematic review: non-adherence and non-persistence in intravitreal treatment. Graefes Arch Clin Exp Ophthalmol.

[10] Talks SJ, Daien V, Mitchell P (2023). The patient voice in neovascular age-related macular degeneration: findings from a qualitative study. Ophthalmol Ther.

[11] Labiris G, Katsanos A, Fanariotis M, Tsirouki T, Pefkianaki M, Chatzoulis D, Tsironi E (2008). Psychometric properties of the Greek version of the NEI-VFQ 25. BMC Ophthalmol.

[12] Marakis TP, Koutsandrea C, Chatzistefanou KI, Tountas Y (2018). Treatment satisfaction of patients with neovascular age-related macular degeneration treated with anti-vascular endothelial growth factor agents. Int Ophthalmol.

[13] Daldal H, Turkyilmaz M, Balikoglu Yilmaz M, Berberoglu U (2021). The effect of ranibizumab loading treatment on vision-related quality of life in diabetic macular edema. Clin Pract.

[14] Garweg JG, Stefanickova J, Hoyng C, Schmelter T, Niesen T, Sowade O, Sivaprasad S (2019). Vision-related quality of life in patients with diabetic macular edema treated with intravitreal aflibercept: the AQUA study. Ophthalmol Retina.

[15] Bertelmann T, Feltgen N, Scheffler M, Hufenbach U, Wiedon A, Wilhelm H, Ziemssen F (2016). Vision-related quality of life in patients receiving intravitreal ranibizumab injections in routine clinical practice: baseline data from the German OCEAN study. Health Qual Life Outcomes.

[16] Morikawa S, Okamoto F, Murakami T, Sugiura Y, Hiraoka T, Okamoto Y, Oshika T (2022). Time course of changes in vision-related quality of life following intravitreal ranibizumab treatment for branch retinal vein occlusion. Sci Rep.

[17] Tan W, Zou J, Yoshida S, Jiang B, Zhou Y (2020). The role of inflammation in age-related macular degeneration. Int J Biol Sci.

[18] Bian W, Wan J, Tan M, Su J, Yuan Y, Wang Z, Li S (2020). Predictors of health-related quality of life in Chinese patients receiving treatment for neovascular age-related macular degeneration: a prospective longitudinal study. BMC Ophthalmol.

[19] Rausch-Koster PT, Rennert KN, Heymans MW, Verbraak FD, van Rens GH, van Nispen RM (2022). Predictors of vision-related quality of life in patients with macular oedema receiving intra-vitreal anti-VEGF treatment. Ophthalmic Physiol Opt.

[20] Okada M, Wong TY, Mitchell P (2021). Defining nonadherence and nonpersistence to anti-vascular endothelial growth factor therapies in neovascular age-related macular degeneration. JAMA Ophthalmol.

[21] Semeraro F, Morescalchi F, Duse S, Parmeggiani F, Gambicorti E, Costagliola C (2013). Aflibercept in wet AMD: specific role and optimal use. Drug Des Devel Ther.

[22] Kolačko Š, Predović J, Tomić A, Oršulić V (2023). Life quality in patients with impaired visual acuity undergoing intravitreal medication applications. Int J Environ Res Public Health.

[23] Matamoros E, Maurel F, Léon N (2015). Quality of life in patients suffering from active exudative age-related macular degeneration: the EQUADE study. Ophthalmologica.

[24] Denys P, Miere A, Colantuono D, Jung C, Souied EH (2022). Intravitreal injections during COVID-19 outbreak: Protective measures, total duration of care and perceived quality of care in a tertiary retina center. Eur J Ophthalmol.

[25] Shahzad H, Mahmood S, McGee S (2023). Non-adherence and non-persistence to intravitreal anti-vascular endothelial growth factor (anti-VEGF) therapy: a systematic review and meta-analysis. Syst Rev.

[26] Holekamp N, Gentile B, Giocanti-Aurégan A (2024). Patient experience survey of anti-vascular endothelial growth factor treatment for neovascular age-related macular degeneration and diabetic macular edema. Ophthalmic Res.

[27] Thinggaard BS, Pedersen M, Sorensen TL, Grauslund J, Stokholm L (2023). Patient perspectives and barriers in the treatment of neovascular age-related macular degeneration in Denmark: a qualitative study. BMJ Open.

[28] Chang MA, Kapre A, Kaufman D (2022). Patient preference and treatment satisfaction with a port delivery system for ranibizumab vs intravitreal injections in patients with neovascular age-related macular degeneration: a randomized clinical trial. JAMA Ophthalmol.

